# Death from a Homœopathic Dose of Chloral

**Published:** 1873-04

**Authors:** 


### Miscellaneous.

### Death from Homœopathic Doses of Chloral.

We find in the Toronto *Globe*, of January 18th, an account of the death, under the combined influence of chloroform, hydrate of chloral and sulphate of morphia, of C. B. Jones, a homœopathic physician. As will be seen by the subjoined evidence, the deceased had submitted to a surgical operation by Duncan Campbell, also a homœopathic physician, who seems on his own admission wholly responsible for the manner in which the patient was treated while recovering from the effects of the anæsthetic. It is clear that death was chiefly, if not entirely, due to the large (homœopathic?) and repeated doses of hydrate of chloral. Duncan Campbell is, we believe, looked upon as a leading homœopathic practitioner in Ontario, inasmuch as his name appears first in the board of five gentlemen of that school who act in conjunction with the council of the College of Physicians and Surgeons of Ontario—

the only body now empowered to issue licenses to practice medicine in that province. The following is his testimony, with that of others, given at the coroner's inquest:

"Duncan Campbell, M. D., sworn, said: 'About three weeks ago deceased called at my house and complained to me of what he took to be internal piles; I told him I thought he was in error as to the nature of his complaint, judging from the symptoms; I said I thought it was fissure of the rectum, and advised him to submit to an operation for its radical cure; he refused at that time, contenting himself with obtaining temporary relief; he consented afterward to have the operation performed, but stipulated that he should not only have chloroform during the operation, but should be kept under the influence of chloral hydrate until the pain should have time to subside. The operation was performed at three o'clock yesterday afternoon; the fissure was large and deep, and must have caused him great pain; I administered chloroform, and the operation was successfully performed; I then gave him forty grains of chloral hydrate, but as this did not bring him relief, I administered a second dose, with a quarter of a grain of sulphate of morphia; I then left, his condition being as good as could possibly be expected; I was recalled in about half an hour, and on arriving found him dead.'

"Lorne Colin Campbell, M. D., sworn, said: 'I was requested by my father, Dr. Campbell, to assist him at an operation on the deceased. Deceased was perfectly conscious after the operation, and insisted that it had not been performed at all. My father weighed out two drachms of chloral hydrate, and administered one-third. Twenty-five minutes after he gave the deceased another dose, combined with a quarter of a grain of the sulphate of morphia. Deceased then went to sleep, and we both withdrew, not apprehending any danger.'

"Thomas Payne, M. D., sworn, said: 'I have heard the evidence of Dr. Campbell and his son, and am of the opinion that the treatment as detailed in the evidence was judicious.'

"After a short deliberation the jury returned the following verdict: 'That the deceased, Charles Blackburn Jones, died on the 15th inst., from the effects of chloral hydrate, after a most painful operation, and it was given at the previous request of the deceased, who purchased the drug himself.'"—*New York Med. Journal.*

